# Association of gender, *ABCA1 gene* polymorphisms and lipid profile in Greek young nurses

**DOI:** 10.1186/1476-511X-11-62

**Published:** 2012-07-09

**Authors:** Vana Kolovou, Apostolia Marvaki, Agathi Karakosta, Georgios Vasilopoulos, Antonia Kalogiani, Sophie Mavrogeni, Dimitrios Degiannis, Christina Marvaki, Genovefa Kolovou

**Affiliations:** 1 Molecular Immunology Laboratory, Onassis Cardiac Surgery Center Athens, Athens, Greece; 2 Thriassio General Hospital, Magoula, Attica, Greece; 3 Department of Nursing, A' Technological Educational Institute of Athens, Athens, Greece; 4 1st Cardiology Department, Onassis Cardiac Surgery Center Athens, Athens, Greece; 5 Onassis Cardiac Surgery Center, 356 Sygrou Ave 176 74, Athens, Greece

## Abstract

**Objective:**

One of the important proteins involved in lipid metabolism is the ATP-binding cassette transporter A1 (ABCA1) encoding by *ABCA1 gene*. In this study we evaluated the single nucleotide polymorphisms (SNPs) of *ABCA1 gene.* We analyzed SNPs in chromosome 9 such as *rs2230806 (R219K)* in the position 107620867*, rs2230808 (R1587K)* in the position 106602625 and *rs4149313 (I883M*) in the position 106626574 according to gender and lipid profile of Greek nurses.

**Methods:**

The study population consisted of 447 (87 men) unrelated nurses who were genotyped for *ABCA1 gene* polymorphisms. Additionally, lipid profile [total cholesterol, triglycerides, high density lipoprotein cholesterol, low density lipoprotein cholesterol (LDL-C) and apolipoprotein A1] was evaluated.

**Results:**

The distribution of all three studied *ABCA1 gene* polymorphisms did not differ according to gender. However, only *R219K genotype* distribution bared borderline statistical significance (p = 0.08) between the two studied groups. Moreover, allele frequencies of *R219K, R1587K* and *I88M* polymorphisms did not differ according to gender. In general, blood lipid levels did not seem to vary according to *ABCA1 gene* polymorphisms, when testing all subjects or when testing only men or only women. However, a significant difference of LDL-C distribution was detected in all subjects according to *R1587K genotype*, indicating lower LDL-C levels with KK polymorphism (p = 0.0025). The above difference was solely detected on female population (p = 0.0053).

**Conclusions:**

The *ABCA1 gene* polymorphisms frequency, distribution and lipid profile did not differ according to gender. However, in the female population the KK genotype of *R1587K gene* indicated lower LDL-C levels. Further studies, involving a higher number of individuals, are required to clarify genes and gender contribution.

## Introduction

ATP-binding cassette transporter A1 (ABCA1) mediates the transport of cholesterol and phospholipids from cells to lipid-poor apolipoproteins. Animals and human studies documented that defects in the ABCA1 pathway are significant determinants of coronary artery disease (CAD) [1]. Inactivation of *ABCA1 gene* in macrophages increases atherosclerotic lesions in hyperlipidemic mice [2,3], and overexpressing human ABCA1 in transgenic mice retards atherogenesis [4,5]. The *ABCA1 gene* is located on the chromosome 9 in the area 9q31.1 and encodes ABCA1 protein. ABCA1 protein is expressed in liver, macrophages, intestines, lungs etc. Several *ABCA1 gene* polymorphisms were identified such as *rs2230806 (R219K)* in the chromosomal position 107620867, *rs2230808 (R1587K)* in the chromosomal position 106602625 and *rs4149313 (I883M*) in the chromosomal position 106626574]. This study in line with our previous work [6] was undergone to evaluate the association of gender, three *ABCA1 gene* polymorphisms mention above and lipid profile in Greek male and female nurses.

## Materials and methods

### Subjects

The genotyping of 447 (87 male) nurse students median age 22 (21–25) years old, who were attended to the University of Nursing of Technological and Educational Institution, was performed. All students had no personal history of CAD and were not taking any drugs. Other exclusion criteria were diabetes mellitus, thyroid and liver disease, high alcohol consumption, professional athleticism and any chronic disease.

All students were attended to the University every day and were staying for 8–10 hours. Students were eating (breakfast, snacks and lunch) at the school canteen which served typical Mediterranean food. Only one meal daily (dinner) was most likely to be different in each student.

The University of Nursing of Technological and Educational Institution Ethics Committee approved the protocol of this study. All subjects signed an informed consent form.

### Blood chemistry

Plasma total cholesterol (TC), triglycerides (TGs), high density lipoprotein cholesterol (HDL-C) and apolipoprotein A1 (Apo A1) were measured using enzymatic colorimetric methods on Roche Integra Biochemical analyzer with commercially available kits (Roche). The serum low density lipoprotein cholesterol (LDL-C) concentration was calculated using the Friedewald formula only in subjects with TGs concentration < 400 mg/dl.

### DNA analysis and determination of blood lipids

The ABCA1 gene polymorphisms (R219K, R1587K and I883M) were detected using polymerase chain reaction (PCR) and restricted fragment length polymorphism analysis (RFLP’s). The PCR was performed using Taq polymerase KAPATaq. The oligonucleotide primers used for R219K and R1587K polymorphisms were described by Saleheen D *et al.*[7] and Tupitsina TV *et al.*[8] respectively.

**Figure 1:**
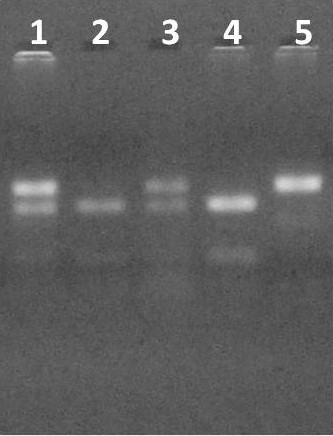
Gel electrophoresis of I883M gene polymorphism

The oligonucleotide primers used for I883M polymorphism were 5’-GAGAAGAGCCACCCTGGTTCCAACCAGAAGAGGAT-3’ and 5’- AGAAAGGCAGGAGACATCGCTT −3 as described by Clee SM *et al.*[9]. PCR was subjected to 95 ^ο^C for 5 min, thirty cycles of 95 ^ο^C for 30 s, 65 ^ο^C for 30 s and 72 ^ο^C for 30 s and final extension to 72 ^ο^C for 7 min, producing a fragment of 132 bp. This fragment was subsequently cleaved by EcorV, creating fragments for I allele 97 bp and 35 bp and for M allele 132 bp, which were subjected to electrophoresis on an agarose gel 4% and visualized with ethidium bromide (Figure 1).

## Statistical analysis

The Shapiro-Wilk test was performed to test for normal distribution of continuous variables. The results are given as median and interquartile range (IQR), whereas, all qualitative variables are presented as absolute or relative frequencies. The Mann–Whitney *U* test or the Fisher’s exact test was employed for comparison of continuous or categorical variables, respectively. The comparison within groups was performed by test for equality of proportions (Bonferroni correction). The Kruskal – Wallis H statistic was employed in order to detect differences in lipid levels according to three different polymorphisms of ABCA1 gene (R219K, R1587K and I883M). ABCA1 polymorphisms’ distribution according to lipid profile was tested using the Kruskal Wallis test in men, in women and in all patients. All results were corrected for multiple testing (Bonferroni correction). All tests were two-tailed and statistical significance was established at 5% (p < 0.05). Data were analysed using Stata ™ (Version 10.1 MP, Stata Corporation, College Station, TX 77845, USA).

## Results

Comparison of various characteristics according to gender showed that the study group was homogenous regarding age (p > 0.05) (Table 1). However, significant differences were detected regarding other anthropometric characteristics and lipidemic profile (Table 1). Specifically, BMI, waist and mean blood pressure measurements were higher in men when compared to women (Table 1). Moreover, TG levels were significantly elevated in men, whereas HDL-C and Apo A1 levels were significantly elevated in women. No differences were detected for TC measurements between men and women (Table 1).

**Table 1:** Characteristics of the study population

	**Men** **(n = 87)**	**Women** **(n = 360)**	**Total** **(n = 447)**	P*
***Anthropometric data***				
Age (years)	21 [20–25]	23 [21–25]	22 [21–25]	0.13
BMI (Kg/m^2^)	24 [22–26]	23 [20–24]	22 [20–25]	<0.001
Waist (cm)	92 [87–97]	87 [80–94]	88 [81–95]	<0.001
Mean BP (mmHg)	87 [80–90]	83 [76.7-90]	87 [80–90]	0.007
***Lipid profile (mg/dl)***				
Total Cholesterol	204 [155–250]	198 [158–238]	198 [158–240]	0.38
Triglycerides	131 [97–185]	89 [61–152]	97 [65–167]	<0.001
HDL cholesterol	59 [46–76]	66 [52–83]	65 [51–82]	0.01
LDL cholesterol	108 [74–149]	105 [76–128]	106 [76–132]	0.25
Apo A1	114 [96–154]	153 [111–189]	147 [107–183]	<0.001
***Other variables***				
Smoking (yes/no) - (%)	48/38 (56%-44%)	127/207 (38%-62%)	175/245 (42%-58%)	0.003
History of CAD (yes/no) - (%)	12/68 (15%-85%)	79/228 (26%-74%)	91/296 (23.5%-76.5%)	0.04

*Comparisons are made according to sex. Statistical tests performed: Mann–Whitney U test or Pearson’s chi-square test were employed for comparison of continuous or categorical data, respectively.

The distribution of all *ABCA1 gene* polymorphisms studied did not differ according to gender (Table 2). However, only R219K distribution bared borderline significance between the two groups studied (Table 2). A post hoc power calculation was conducted employing the calculated differences in the distribution of R219K polymorphism between men and women. The post hoc power (<0.6) was not sufficient enough to support the hypothesis. Furthermore, none of the comparisons within groups (concerning the rest of the polymorphisms) were statistically significant (p > 0.1). Moreover, allele frequencies of *R219K, R1587K* and *I88M* polymorphisms did not differ according to gender (Table 2).

**Table 2:** Distribution of R219K, R1587K and I883M polymorphisms and allele frequencies according to sex

	**Men** **(n = 87)**	**Women** **(n = 360)**	**P**
**R219K**			
RR	39 (17.3%)	186 (82.7%)	0.08*
RK	45 (23.8%)	144 (76.2%)	
KK	3(9.4%)	29 (90.6%)	
**R1587K**			
RR	37 (17.5%)	174 (82.5%)	0.12*
RK	45 (23.5%)	146 (76.5%)	
KK	5 (11.4%)	39 (88.6%)	
**I883M**			
II	64 (20.7%)	245 (79.3%)	0.66*
IM	22 (17.2%)	106 (82.8%)	
MM	1 (12.5%)	7 (87.5 %)	
**Allele frequencies for R219K polymorphism**			
R allele frequency	0.71	0.72	0.85**
K allele frequency	0.29	0.28	
**Allele frequencies for R1587K polymorphism**			
R allele frequency	0.68	0.69	0.85**
K allele frequency	0.32	0.31	
**Allele frequencies for I883M polymorphism**			
I allele frequency	0.86	0.83	0.4**
M allele frequency	0.17	0.14	

Fisher’s exact test - **Test for equality of proportions.

In general, blood lipid levels did not seem to vary according to *ABCA1 gene* polymorphisms, when testing all subjects (Table 3) or when testing only men (Table 4) or only women (Table 5). However, a significant difference of LDL-C distribution was detected in all patients according to *R1587K genotype*, indicating lower LDL-C levels with KK polymorphism (Table 3). The above difference was solely detected on female population (Table 5).

**Table 3:** Blood lipid levels according to ABCA1 polymorphisms in all subjects

***ALL SUBJECTS (n = 447)***	**Genotype**	**Median**	**IQR**	**P**^*†*^
***R219K***				
***Total cholesterol (mg/dl)***	RR	199	[160 – 239]	0.74
	RK	194	[158 – 241]	
	KK	205	[160–244]	
***Triglycerides (mg/dl)***	RR	98	[65 – 162]	0.66
	RK	98	[63 – 170]	
	KK	95	[69 – 140]	
***HDL cholesterol (mg/dl)***	RR	66	[51 – 80]	0.94
	RK	64	[52.5 – 84]	
	KK	63	[50.5 – 85]	
***LDL cholesterol (mg/dl)***	RR	106	[78 – 131]	0.18
	RK	104	[71.5 - 131]	
	KK	118	[92–147]	
***Apo A1 (mg/dl)***	RR	139	[108 – 180]	0.09
	RK	152	[106–187]	
	KK	164	[115–202]	
***R1587K***				
***Total cholesterol (mg/dl)***	RR	190	[145 – 238]	0.08
	RK	203	[170 – 241]	
	KK	199	[158 – 222]	
***Triglycerides (mg/dl)***	RR	95	[60 – 167]	0.35
	RK	103	[68 – 168]	
	KK	85	[69–150]	
***HDL cholesterol (mg/dl)***	RR	65	[51 – 81]	0.73
	RK	65	[53 – 83]	
	KK	62	[47 – 85]	
***LDL cholesterol (mg/dl)***	RR	100	[66 – 122]	0.0025
	RK	114	[84 –139]	
	KK	88	[87 – 89]	
***Apo A1 (mg/dl)***	RR	145	[104 – 185]	0.78
	RK	148	[109 – 180]	
	KK	147	[107 – 194]	
***I883M***				
***Total cholesterol (mg/dl)***	II	198	[155 – 240]	0.74
	IM	198	[164 – 238]	
	MM	197	[175 – 253]	
***Triglycerides (mg/dl)***	II	99	[65 – 163]	0.49
	IM	91	[62 – 171]	
	MM	170	[78 – 233]	
***HDL cholesterol (mg/dl)***	II	65	[51 – 82]	0.83
	IM	64	[52 – 82]	
	MM	68	[55 – 95]	
***LDL cholesterol (mg/dl)***	II	105	[74 – 130]	0.52
	IM	108	[81 – 133]	
	MM	117	[64 – 132]	
***Apo A1 (mg/dl)***	II	143	[104 – 182]	0.62
	IM	148	[109 – 184]	
	MM	153	[111 – 200]	

*HDL: high density lipoprotein, LDL: low density lipoprotein, Apo A1: apolipoprotein A1.*^*†*^*P values among genotypes from Kruskal Wallis test performance.*

**Table 4:** Blood lipid levels according to ABCA1 polymorphisms in men

***MEN (n = 87)***	**Genotype**	**Median**	**IQR**	**P**^*†*^
***R219K***				
***Total cholesterol (mg/dl)***	RR	199	[149 – 250]	0.86
	RK	205	[164 – 258]	
	KK	222	[122 – 240]	
***Triglycerides (mg/dl)***	RR	125	[100 – 177]	0.58
	RK	142	[97 – 196]	
	KK	134	[50 – 163]	
***HDL cholesterol (mg/dl)***	RR	61	[49 – 74]	0.28
	RK	57	[46 – 81]	
	KK	46	[45 – 48]	
***LDL cholesterol (mg/dl)***	RR	109	[66 – 146]	0.84
	RK	106	[76 – 150]	
	KK	149	[67 – 159]	
***Apo A1 (mg/dl)***	RR	112	[100 – 135]	0.58
	RK	117	[95 – 168]	
	KK	116	[81 – 180]	
***R1587K***				
***Total cholesterol (mg/dl)***	RR	192	[146 – 273]	0.28
	RK	206	[166 – 241]	
	KK	122	[110 – 205]	
***Triglycerides (mg/dl)***	RR	131	[79 – 199]	0.92
	RK	131	[100 – 176]	
	KK	153	[108 – 170]	
***HDL cholesterol (mg/dl)***	RR	60	[51 – 74]	0.41
	RK	58	[48 – 76]	
	KK	45	[42 – 48]	
***LDL cholesterol (mg/dl)***	RR	99	[70 – 150]	0.16
	RK	119	[81 – 149]	
	KK	67	[58–106]	
***Apo A1 (mg/dl)***	RR	114	[94 – 156]	0.79
	RK	116	[100 – 149]	
	KK	100	[96 – 116]	
***I883M***				
***Total cholesterol (mg/dl)***	II	201	[148 – 255]	0.27
	IM	214	[180 – 241]	
	MM	120	-	
***Triglycerides (mg/dl)***	II	126	[84–177]	0.18
	IM	169	[118 – 250]	
	MM	152	-	
***HDL cholesterol (mg/dl)***	II	59	[47 – 75]	0.44
	IM	57	[48 – 78]	
	MM	41	-	
***LDL cholesterol (mg/dl)***	II	111	[70–148]	0.39
	IM	105	[80 – 149]	
	MM	49	-	
***Apo A1 (mg/dl)***	II	110	[94 – 148]	0.14
	IM	148	[107 – 169]	
	MM	112	-	

*HDL: high density lipoprotein, LDL: low density lipoprotein, Apo A1: apolipoprotein A1.*^*†*^*P values among genotypes from Kruskal Wallis test performance.*

**Table 5:** Blood lipid levels according to ABCA1 polymorphisms in women

***WOMEN (n = 360)***	**Genotype**	**Median**	**IQR**	**P**^*†*^
***R219K***				
***Total cholesterol (mg/dl)***	RR	200	[160 – 238]	0.64
	RK	193	[155 – 238]	
	KK	204	[163 – 249]	
***Triglycerides (mg/dl)***	RR	89	[57 – 152]	0.88
	RK	87	[61 – 159]	
	KK	94	[70 – 134]	
***HDL cholesterol (mg/dl)***	RR	66	[52 – 82]	0.84
	RK	65	[55 – 85]	
	KK	66	[55 – 85]	
***LDL cholesterol (mg/dl)***	RR	105	[79 – 127]	0.18
	RK	104	[70 – 126]	
	KK	117	[93 – 141]	
***Apo A1 (mg/dl)***	RR	145	[111 – 187]	0.16
	RK	155	[110 – 194]	
	KK	164	[122 – 204]	
***R1587K***				
***Total cholesterol (mg/dl)***	RR	189	[145 – 231]	0.09
	RK	203	[172 – 240]	
	KK	203	[163 – 224]	
***Triglycerides (mg/dl)***	RR	87	[57 – 146]	0.61
	RK	96	[61 – 160]	
	KK	81	[69 – 136]	
***HDL cholesterol (mg/dl)***	RR	66	[51 – 82]	0.82
	RK	67	[55 – 84]	
	KK	64	[52 – 86]	
***LDL cholesterol (mg/dl)***	RR	100	[66 – 121]	0.0053
	RK	112	[85 – 132]	
	KK	106	[83 – 133]	
***Apo A1 (mg/dl)***	RR	152	[109 – 189]	0.59
	RK	155	[114 – 188]	
	KK	153	[116 – 194]	
***I883M***				
***Total cholesterol (mg/dl)***	II	198	[156 – 237]	0.54
	IM	196	[160 – 238]	
	MM	214	[181 – 273]	
***Triglycerides (mg/dl)***	II	90	[61–155]	0.45
	IM	86	[58 – 148]	
	MM	188	[73 – 274]	
***HDL cholesterol (mg/dl)***	II	66	[52–82.5]	0.61
	IM	66	[53 – 83]	
	MM	73	[60 – 111]	
***LDL cholesterol (mg/dl)***	II	104	[74 – 125]	0.34
	IM	109	[83 – 132]	
	MM	124	[66 – 134]	
***Apo A1 (mg/dl)***	II	154	[111–189]	0.86
	IM	149	[110 – 188]	
	MM	166	[110 – 226]	

*HDL: high density lipoprotein, LDL: low density lipoprotein, Apo A1: apolipoprotein A1.*^*†*^*P values among genotypes from Kruskal Wallis test performance.*

## Discussion

In this study we evaluated the association of gender, *ABCA1 gene* polymorphisms and lipid profile in Greek young nurses.

In previous study we evaluated the influence of *ABCA1 gene* polymorphisms on lipid profile in female Greek nurses. In the present study, we assessed more subjects, included men and evaluated one more polymorphism, namely *rs4149313 (I883M)*.

### R219K polymorphism

One of the first study which evaluated the R219K polymorphism and lipid profile in man was published by Clee SM *et al.*[9]. They genotyped 804 Dutch men with CAD who were participated in the Regression Growth Evaluation Statin Study (REGRESS). The frequency of K allele in their study was similar to our study (25.4% and 28%, respectively). They found that K allele carriers had decreased progression of atherosclerosis and a reduced risk of coronary events. Furthermore, the K allele was associated with lower plasma TGs concentration and a trend toward higher HDL-C concentration. Since then, many other studies correlated the R219K variant with the lipid profile and risk for CAD in various populations and ethnicities [6,9-16]. Conversely, Pasdar *et al*. [10] did not support a major role of the *ABCA1 gene* as risk factor for ischemic stroke. Srinivasan *et al.*[11] found that the K219 allele frequency differs markedly between blacks and whites, and that the variant-allele modulates the association between age and HDL-C, as well as body fatness and TGs concentration in a beneficial manner only in whites. Bertolini *et al*. [12] found that in subjects with CAD, the prevalence of RK and KK genotypes was lower than in subjects free of CAD (33.0% versus 51.5%). Zhao *et al*. [13] found a significant upward trend in the sequence of RR, RK, and KK genotypes with a significant difference between RR genotype (50.5 ± 15.6 mg/dl, 1.3 mmol/l ± 0.4 mmol/l) and KK genotype (54.6 ± 15.6 mg/dl, 1.4 mmol/l ± 0.4 mmol/l), especially in males. This was not proved by our previous study where only women were participated [6]. Additionally, Frikke-Schmidt *et al.*[14] did not find any association with HDL-C levels in Danish population. Kitjaroentham *et al.*[15] studied overweight/obese Thai subjects of both sexes. The overweight/obese men who had the K allele had lower HDL-C than controls. In our study, we do not find any association of the R219K variant with any parameter of the lipid profile. Also, the distribution of all studied *ABCA1 gene* polymorphisms was not different according to gender. Noteworthy to mention is that the R219K distribution bared borderline statistical significance (p = 0.08) between two studied groups. In general, blood lipid levels did not seem to vary according to *ABCA1 gene* polymorphisms, when testing all subjects or when testing only men or only women.

### R1587K polymorphism

Clee *et al.*[9] in the study already mention above found that K carriers of R1587K (RK, KK) had lower HDL-C concentration compared with noncarriers in an allele dose-dependent trend. On multiple regression analysis including age, BMI, smoking, and TG as covariates, the R1587K genotype remained a significant predictor of HDL-C. Frikke-Schmidt et al. [16] associated the R1587K variant with a stepwise decrease in HDL-C in women in heterozygotes and homozygotes, and with a similar trend in men. Mantaring *et al.*[17] did not find any statistical significance between R1587K variant with the lipid profile. However, Tsai *et al.*[18] found that after fenofibrate treatment the KK genotype of R1587K was associated with significantly increased small HDL and with increased HDL particle concentrations. In our previous study [6] where only women were participated, the difference in TC, LDL-C and TGs concentration was detected between RK and RR genotypes. In this study we found according to gender that blood lipid levels did not seem to vary in genotypes of R1587K polymorphism. However, a significant difference of LDL-C distribution was detected in female population. We also expected to found a difference in TC and TGs values between genotypes of female population as in previous study [6]. However, in the current study, in order to expand the study population, we included women with different lipid profiles, affecting the overall variability and altering the final results. Generally, lipid profile can be influenced by several genetic and environmental factors such as smoking status, eating habits, associated customs (fasting periods), sedentary life style, body composition, gender and others. The most important factors influencing the lipid profile include the sex hormone status and age. Unfortunately, in our study apart from age, smoking status and BMI we have not gone to all other details which potentially could influenced the results (added data consisted of 1/6 of women’s study cohort).

### I883M polymorphism

Tan *et al.*[19] studied the I883M variant in Malays and Chinese population. They found an association with lipoprotein(a) concentration in Malays population and with apolipoprotein B concentration in Chinese population. However, Clee *et al.*[9] did not find any difference according to lipid levels and genotypes in carriers of the I883M, although individuals with MM genotype had higher progression in minimum obstruction diameter and cardiac event rate compared with the II genotype individuals. Hodoğlugil *et al.*[20] correlated the I883M variant with higher HDL-C concentration in both sexes. Similarly, Jensen *et al.*[21] among younger women and Porchay-Baldérelli *et al.*[22] in population with type 2 diabetes mellitus found that the M allele of I883M was associated with higher HDL-C concentration. Additionally, Mantaring *et al.*[17] found some differences in allele frequency of *I883M gene*, which were also present between the highest and lowest HDL-C concentration groups (36% vs 20%; *P*_trend_ = 0.05). On the contrary, Kitjaroentham *et al.*[15] did not find any difference in HDL-C concentrations among I883M genotype polymorphism. Although, this variant was common in Thai ethnic groups, there were no differences detected in genotype frequency between overweight/obese and control subjects groups. Similarly, Frikke-Schmidt *et al.*[14] found that I883M did not affect HDL-C levels but predicted higher risk of CAD. Also, Sandhofer *et al.*[23] found that I883M variant showed no effect on plasma lipids or carotid atherosclerosis. In our study, we have not found any association with the lipid profile. However, there are some data from Delgado-Lista *et al.*[24] suggesting that the major allele homozygotes for *ABCA1 gene* polymorphisms i27943 (rs2575875) and R219K (rs2230806) have a lower postprandial response as compared to minor allele carriers. The fact that in current study blood lipid levels did not seem to vary according to *ABCA1 gene* polymorphism could be influence, at least in part, because they were measured in the fasting state.

A limitation of this study is the relatively small number of men’s group. However, the effort was put for sample to be homogenous, living in the similar conditions as it happened with our study population.

Another limitation of this type of study is that, studies based on the candidate-gene approach, which have been demonstrated genotype-phenotype associations, are not always replicable.

There are only few studies which compare the influence of *ABCA1 gene* polymorphisms on lipid profile in accordance to gender. The *ABCA1 gene* polymorphisms frequency, distribution and lipid profile did not differ according to gender. However, in the female population the KK genotype of *R1587K gene* indicated lower LDL-C levels.

## Competing interests

The authors declare that they have no competing interests.

## Authors’ contributions

VK participated in the development of hypothesis, drafting of the manuscript and carried out the genetic analysis, AM participated in the molecular genetic studies, AK performed the statistical analysis and drafting of the manuscript, GV and AK collected the blood samples, SM participated in the drafting and revising the manuscript, DD participated in revising the manuscript critically for important intellectual content, CM participated in the study design and its coordination and GK conceived the study and participated in the development of the hypothesis, the study design and drafting of the manuscript. All authors read and approved the final manuscript.
